# Supplementary material for: Mice Generated with Induced Pluripotent Stem Cells Derived from Mucosal-Associated Invariant T Cells
Source: Biomedicines. 2024 Jan 9;12(1):137. doi: 10.3390/biomedicines12010137 (PMC10813358; doi:10.3390/biomedicines12010137)
Supplement: Supplementary file 1 [file biomedicines-12-00137-s001.zip › Figure S1rev.pdf]

Figure S1

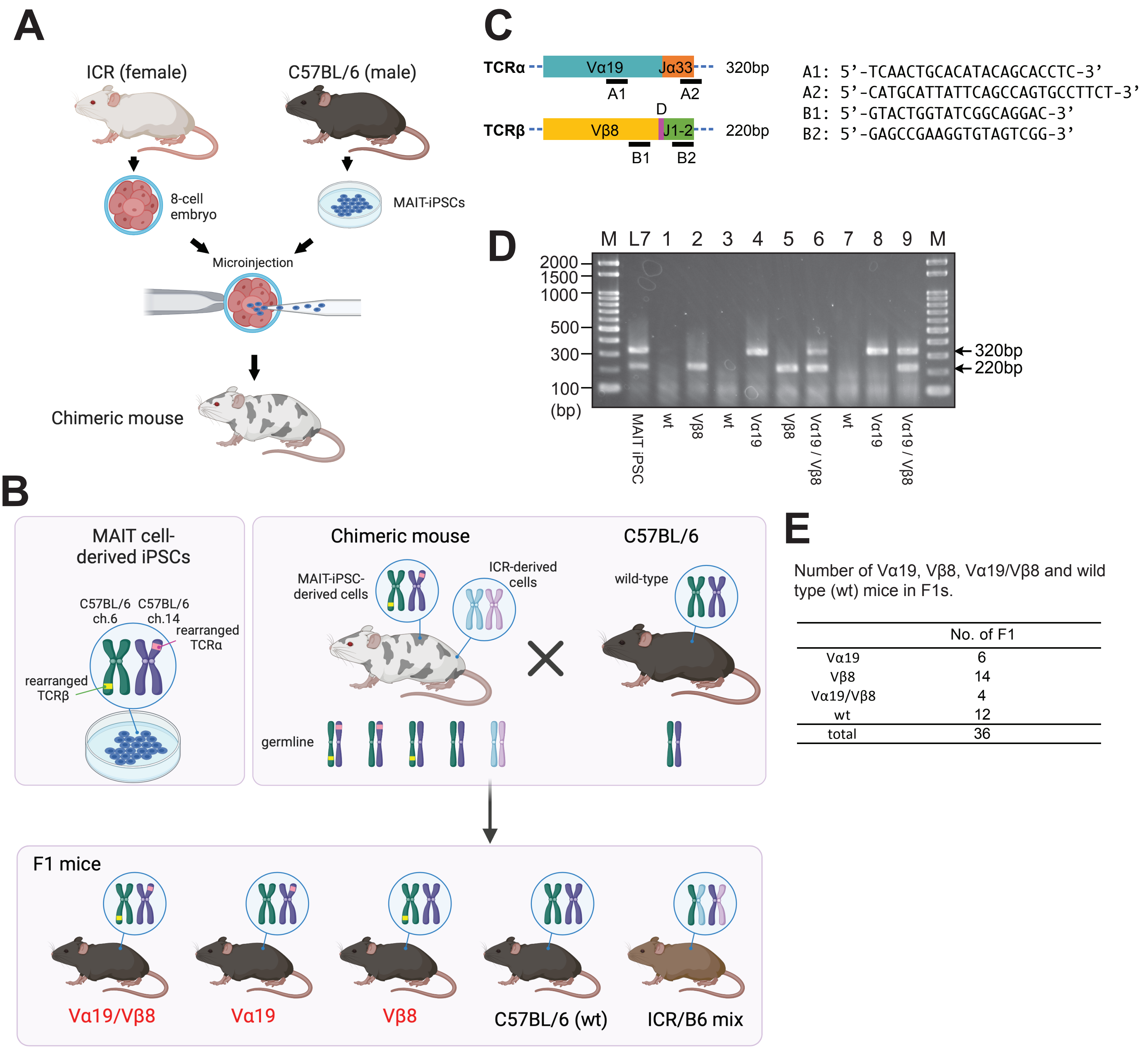

Figure S1 (related to Figure 1) Generation of C57BL/6 (B6) mice harboring rearranged TCR loci specific for MAIT cells. (A) Generation of chimeric mice with iPSCs derived from MAIT cells (MAIT-iPSCs) MAIT-iPSCs established from male B6 mice were microinjected into ICR mouse zygotes, and resultant zygotes were implanted into the pseudopregnant ICR mice to obtain chimeric mice. **(B)** TCR gene locus configuration in F1 progenies from the chimeric mouse Genes encoding TRAV (TCRα) and TRBV (TCRβ) are located in chromosomes (ch.) 14 and ch.6, respectively. Rearranged TCR configuration (depicted as TCRα and/or TCRβ in the left upper panel) is independently passed on to offspring from chimeric mice upon crossing with B6, resulting in five possible combinations. F1 progenies comprise mice harboring rearranged TCRα and TCRβ loci (designated Vα19/Vβ8 mice), rearranged TCRα locus (designated Vα19 mice), rearranged TCRβ locus (designated Vβ8 mice), C57BL/6, or ICR/B6 mix. **(C)** Schematic representation of the rearranged *Trav1*(Vα19)-*Traj33* (Jα33) and *Trbv13-3* (TCRβ8)-*Trvd-Trvj1-2* (J1-2) in the alle of MAIT-iPSC The position and sequences of the primer sets detecting the configurations are indicated. **(D)** Representative PCR results distinguishing each strain MAIT-iPSC served as positive control for both rearranged TCRα and TCRβ shown in (B). (E) A summary of the crossing between the chimeric mouse generated from MAIT-iPSC clone L7-1 (#26) and C57BL/6 females (B6 wt) is shown. The F1s with black coat color were performed PCR genotyping and the number of them carrying the indicated genotypes is depicted. (A) and (B) were created with BioRender.com.
