# Supplementary material for: Mice Generated with Induced Pluripotent Stem Cells Derived from Mucosal-Associated Invariant T Cells
Source: Biomedicines. 2024 Jan 9;12(1):137. doi: 10.3390/biomedicines12010137 (PMC10813358; doi:10.3390/biomedicines12010137)
Supplement: Supplementary file 1 [file biomedicines-12-00137-s001.zip › Figure S2.pdf]

Figure S2

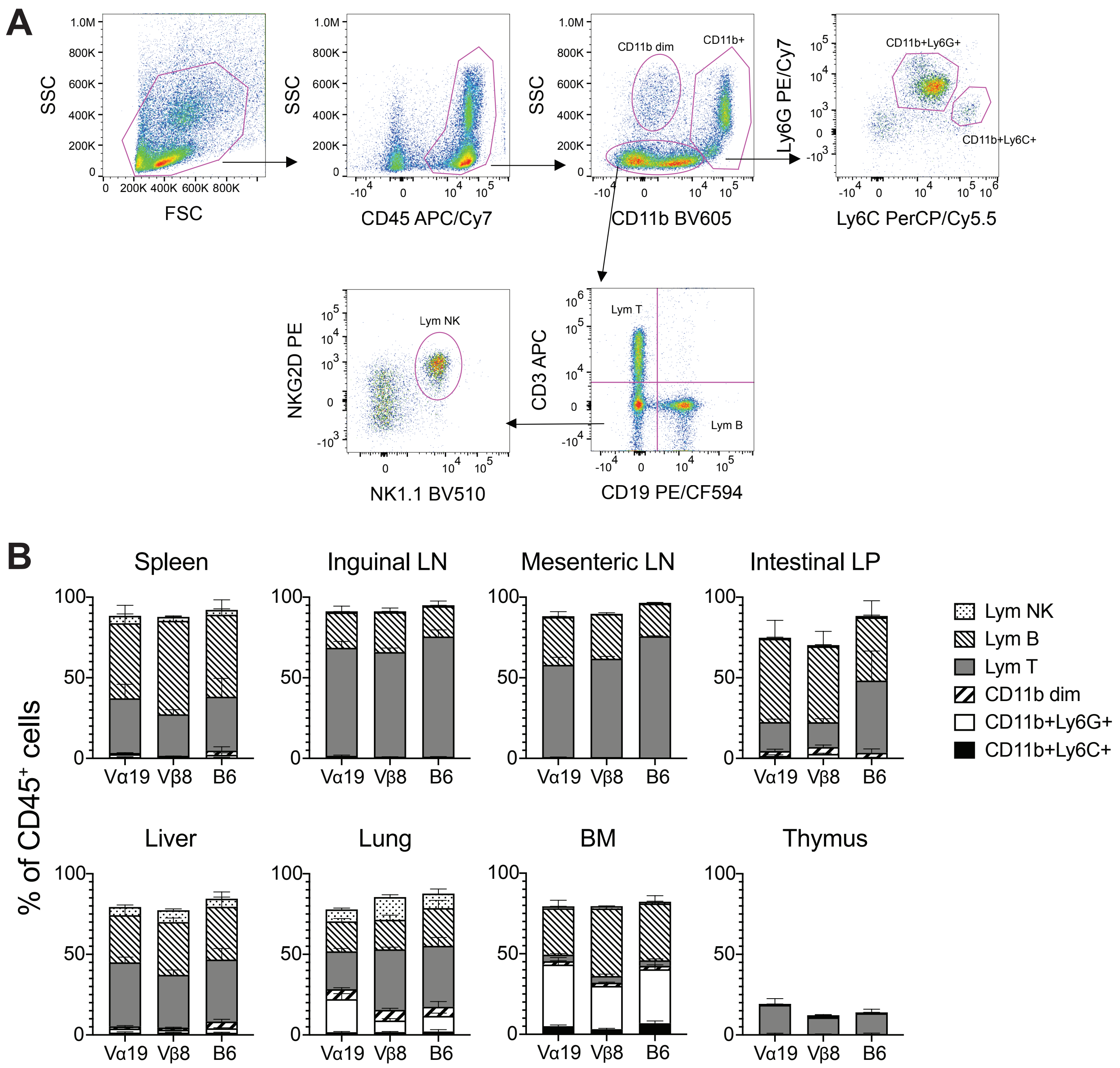

Figure S2 (related to Figure 2) Various leukocyte populations in tissues from Vα19 and Vβ8 mice

(A) Gating strategy to identify macrophage, neutrophil, monocytes, NK cells, and T cells. Isolated cells from mouse tissues were analyzed by flow cytometry with the indicated antibodies. $CD45^+CD11b^{dim}$ cells were identified as macrophages. $CD11b^+$ cells were classified into $CD11b^+Ly6G^+$ (neutrophils) and $CD11b^+Ly6G^+$ (monocytes). CD11b negative population was further divided according to the expression of CD3 and CD19. T cells (Lym T) were defined as $CD3^+CD19^-$, while B cells (Lym B) were CD3-CD19+. CD3-CD19- population was further explored by expression of NKG2D and NK1.1 and the $NKG2D^+NK1.1^+$ cells were identified as NK cells (Lym NK). (B) Relative frequency of NK, B, and T cells, macrophage, neutrophil, and monocytes in tissues from mice. Relative frequency of each subset in the organs is indicated in the graph bar. Data are representative of three independent experiments with a similar tendency. Vα19 mice (Vα19), Vβ8 mice (Vβ8), and control mice (B6).
