# Supplementary material for: Mice Generated with Induced Pluripotent Stem Cells Derived from Mucosal-Associated Invariant T Cells
Source: Biomedicines. 2024 Jan 9;12(1):137. doi: 10.3390/biomedicines12010137 (PMC10813358; doi:10.3390/biomedicines12010137)
Supplement: Supplementary file 1 [file biomedicines-12-00137-s001.zip › Figure S3.pdf]

Figure S3

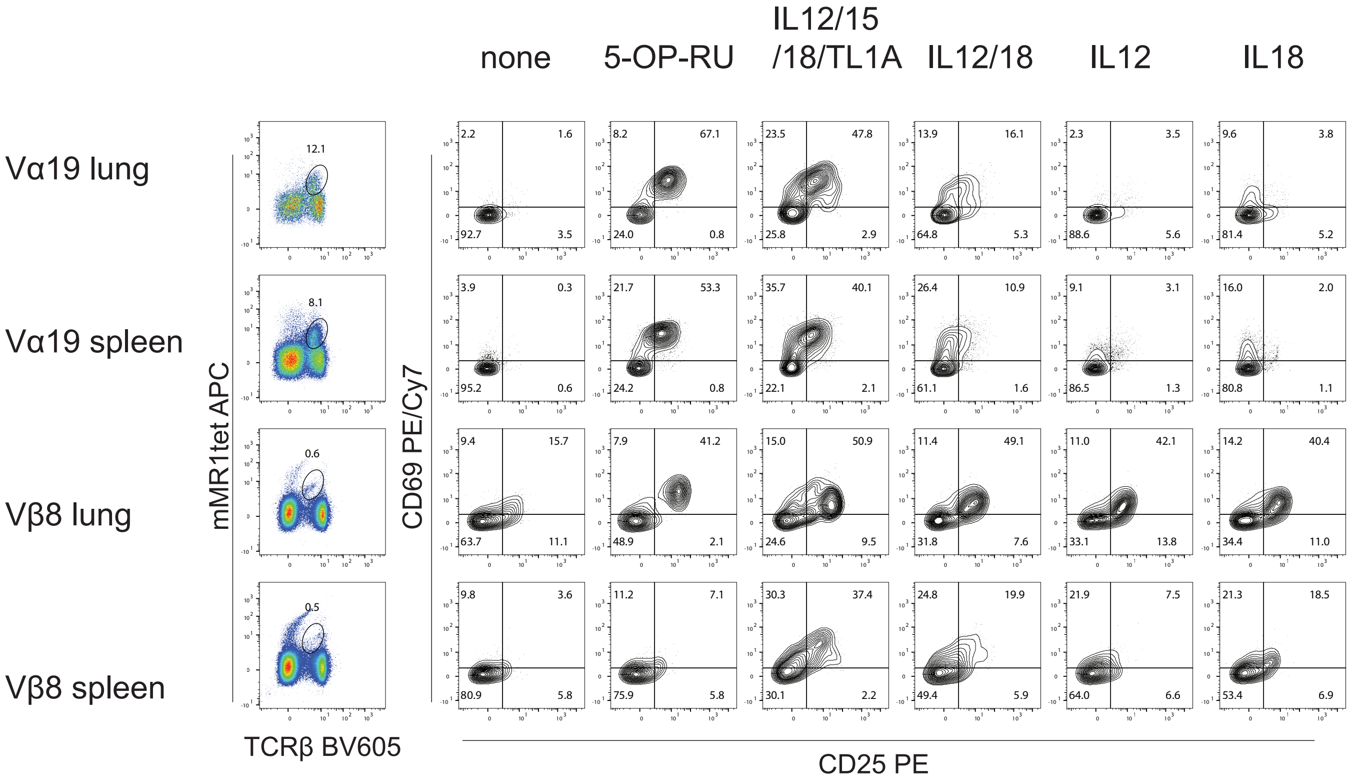

Figure S3 (related to Figure 5) MAIT cells activation induced by an agonist and cytokine(s). Flow cytometric profiles of CD25 and CD69 expression in MAIT cells (TCRβ⁺MR1-tet⁺ cells, the right panels) are shown. Stimuli are indicated above the panels. Data are representative from three independent experiments with a similar profile.
